# Health workforce policy in the Russian Federation: How to overcome a shortage of physicians?

**DOI:** 10.3389/fpubh.2022.1023845

**Published:** 2022-10-20

**Authors:** Igor Sheiman

**Affiliations:** National Research University Higher School of Economics, Moscow, Russia

**Keywords:** workforce, physician shortage, workforce planning, primary health care, post-graduate training, Russian Federation

## Abstract

Russia looks for ways to overcome a shortage of physicians. Health workforce policy is focused on training an additional number of physicians. The current efforts have reduced some areas of the shortage but failed to solve the problem due to many factors that reproduce the deficit. A distorted structure of service delivery with weak primary care generates demand for outpatient specialists and hospital doctors and requires a perpetual increase in their number. The lack of long-term labor planning results in the oversupply of some specialties and the shortage of others. The regulation of post-graduate training is not enough to improve the allocation of physicians across specialties and health system sectors. We argue that an extensive increase in the number of physicians without changing their composition will hardly change the situation. A more active structural policy is required with a focus on strengthening primary care and improving planning and regulation of health workforce structure.

## Introduction

The ability of health systems to respond to new challenges is heavily dependent on the deployment of an adequate supply of health professionals in sufficient numbers, operating in the right areas of service delivery, and with appropriate scope for professional development. This is particularly true for many post-Soviet countries that face inadequate health funding and the legacy of the “Semashko” model. Russia is among the world leaders in the physicians-population ratio: 4.2 physicians per 1,000 population vs. the average of 3.6 for OECD countries (1). However, the country now faces a serious problem of physician shortage, particularly in primary care. The Federal Ministry of Health (MoH) reports that around 20% of physicians' positions in polyclinics were unfilled in 2019 (2).

The coexistence of a relatively high number of physicians and their shortage is a phenomenon that can be accounted to many factors. Partly, this is the result of special health care needs due to country-specific factors such as harsh climate in many regions, low density of large rural population, and high incidence of cardiovascular diseases and accidents (3). The major causes for the Russian health worker supply imbalance, however, are evident in our former research (4) that indicates the important contribution of a deficit of primary healthcare physicians, unequal income opportunities for certain physician specialties, physicians are not adequately supported by nurses, and allied health personnel. The paradoxical situation of “too many too few” requires a special set of workforce policy interventions. Their analysis is relevant for countries that face similar problems.

Following the break-up of the Soviet Union in 1991, the Russian health system has undergone a significant transition to mandatory health insurance (MHI) but has retained its chronic underfunding. Public health expenditures have not exceeded 3.5% of GDP over the last decades (5). The institutional structure of service delivery has not changed much. Most of the facilities are state-owned. Primary health care (PHC) in urban areas is provided by multispecialty polyclinics—separate clinics for adults and children; each has a catchment area and a patient list managed by district therapists, district pediatricians, and general practitioners (GPs)—all of which are collectively referred to as ‘district physicians' (DPs). The catchment population of urban polyclinics ranges from 30,000 to 120,000 people. Big entities employ 15–20 categories of specialists. According to the legislation, PHC is practically the equivalent of outpatient care. Hospitals vary in size, the structure of specialties, and the number of patients (6).

To cope with the problem of physician shortage, the government has started a number of policy activities. The presidential decree of May 2012 set the task to increase the salary of physicians to the level of 200% of the average remuneration in the economy of the corresponding regions, and the salary of nurses to 100%. These targets have been reached in most of 85 regions of the country (7). But this important measure was not enough to eliminate the shortage. Additional measures have been taken, of which the most important is a national program “Providing medical organizations with qualified personnel” (further program) for 2018–2024 (2).

The objective of this paper is to explore the activities and outcomes of this program and some other policy activities. The major research questions: What are the major policy interventions? What are the factors driving the health labor imbalances? What should be done to solve this problem? We analyze the main developments over the last 10 years with a focus on the period of the program implementation.

The analysis is based on a review of the Russian and international literature, as well as materials of the federal and regional health authorities. The official statistical data are supplemented by our estimates and comparisons with OECD countries.

## Policy options and implications

### The program major activities

The major objective of the program is to reduce the shortage of physicians and nurses with focus on primary care. While the government recognizes the existence of a critical imbalance in the health workforce, there is an inadequate study and debate on how best to improve the scope and impact of the program's interventions and investments.

The program is focused on increasing the number of physicians. The admission of students to medical universities increased from 2018/2019 to 2020/2021 by 20% in general medicine specialties [(8), p. 117]. It is expected to raise the number of physicians in state medical organizations from 37.4 per 10,000 population in 2017 to 40.7 by 2024 (Table 1).

**Table 1:** Indicators and targets of the federal program “Providing medical organizations with qualified personnel” in Russia, 2017–2024.

	**2017**	**2019**	**2020**	**2021**	**2022**	**2023**	**2024**
Physicians working in state medical organizations (persons per 10,000 population)	37.4	37.9	38.4	38.9	39.5	40.1	40.7
Medical nurses working in state medical organizations (persons per 10,000 population)	86.2	86.8	87.8	89.1	90.4	92.3	95.1
Outpatient care physicians working in state medical organizations (persons per 10,000 population)	20.7	20.9	21.1	21.4	21.7	22.9	22.5
Per cent of filled physicians positions in units providing outpatient care (individuals with a multiple job-holding coefficient of 1.2)	79.7	81.0	83.0	86.0	89.0	92.0	95.0
Per cent of filled nurses positions in units providing outpatient care (individuals with a multiple job-holding coefficient of 1.2)	88.8	90.0	91.0	92.0	93.0	94.0	95.0
The share of outpatient care physicians in the total number of physicians, %^*^	55.3	55.2	55.0	55.0	55.1	55.2	55.3
Medical nurses-physicians ratio^*^	2.3	2.3	2.3	2.3	2.3	2.3	2.3

* The author's calculation based on the first lines of this table.

Source: (2).

To achieve these indicators, a so-called “target enrollment” of students is expanding. It provides for the involvement of regional governments in the admission of students to medical universities, including post-graduate training. Regional policymakers are supposed to assess the demand for specific medical specialists in their regions, contract universities, and make commitments to financial subsidies for the education of students and the employment of graduates. The share of such targeted enrollments in the total number of admissions to medical universities is to increase from 57% in 2018 to 62% by 2024 (9).

Policies to facilitate health worker employment are being implemented with regional health employment centers established. These centers are to search for medical personnel in the labor market, attract physicians from other regions, and collect information on job vacancies for public distribution. The centers are also to promote the development of a so-called “shift method”, that is the employment of health workers for temporary work in local areas with an acute shortage of medical personnel (10).

To attract medical professionals, some financial benefits have been introduced, including a partial compensation of utilities. Since 2012, a special program has been implemented in rural areas and small towns. Physicians who choose the work in rural settings are provided with a lump sum to buy a house or an apartment.

To improve the quality of medical personnel qualifications, new professional standards and accreditation procedures are being introduced. They are to cover all medical workers in 2022. On contrary to this objective, the so-called “simplified accreditation” of medical university graduates was introduced in 2016. They are allowed to work as district therapists and pediatricians without passing post-graduate training—an approach unknown internationally (6).

### Some outcomes indicators

These several policy interventions yield some important progress, but much more is to be accomplished. The number of physicians fell in 2010–2017 and then increased by 4.6% in 2018–2020. Physician–population ratio had the same trend. The increase in the last years was the result of additional enrollment of medical students. However, the supply of nurses had a downward tendency—both in absolute and relative terms (Table 2).

**Table 2:** Physicians and nurses in Russia, 2010–2020.

	**2010**	**2015**	**2018**	**2019**	**2020**
Physicians, thousands	716	673	704	715	737
Physicians per 10,000 of population	50.1	45.9	47.9	48.7	50.4
Nurses, thousands	1,509	1,550	1,491	1,491	1,490
Nurses per 10,000 of population	105.6	105.8	101.6	101.6	102.0

Source: [(8), p. 114, 116].

To assess the shortage, the program established an indicator of staffing full-time positions of physicians and nurses. It is planned to increase the share of filled outpatient physician positions from 79.7% in 2017 to 95% by 2024. Nearly the same progress is planned for nurses (Table 1).

Using this indicator, the MoH estimates a 39% decrease in the total shortage of physicians and a 37% decrease in the shortage of outpatient physicians over 2016–2019. The size of the latter in 2019 (before the start of the pandemic) was estimated at the level of 8.4%. Among the most wanted outpatient specialties are dentists, radiologists, orthopedic dentists, dermatologists, neurologists, surgeons, and ophthalmologists (2).

The declared strategy of PHC priority is not being realized. The total number of district physicians fell in the period 2010–2017 but then increased only by 1.1% in the period 2018–2021 (Table 3)—partly due to the influx of graduates from medical universities without post-graduate training. As a result, their capacity for high-quality health outcomes is constrained.

**Table 3:** District physicians in Russia, 2010–2021.

		**2010**	**2015**	**2018**	**2020**	**2021**
1	District therapists, thousands	37,835	35,442	36,215	37,380	38,406
2	District pediatricians, thousands	26,723	25,932	28,161	28,722	28,416
3	General practitioners, thousands	8,983	9,520	11,358	10,505	9,839
4	Total number of district physicians, thousands. (1+2+3)	73,541	70,894	75,734	76,607	76,661
5	Total number of physicians in the system managed by the MoH, thousands	625,671	543,604	551,502	557,303	563,608
6	Share of district physicians in total number of physicians,% (4:5)	11.7	13.0	13.7	13.7	13.6
7	Share of GPs in total number of district physicians, % (3:4)	12.2	13.4	14.9	13.7	12.8

Sources: Author's estimates based on (8).

The COVID-19 pandemic has aggravated the shortage of PHC physicians and limited their accessibility. The number of vacant doctor positions doubled in 2021 (11). According to a national population survey in October 2021, 70.1% of respondents reported “the inability to make an appointment with the doctor at first attempt” (12). The pandemic has revealed additional labor shortages, including the lack of infectious disease specialists, rehabilitators, nurses, and social workers. The qualification of many DPs was not enough to manage new cases. The government has mobilized hospital doctors. They took on the major burden of the pandemic.

In the hospital care sector, there is an oversupply of physicians. According to the official estimate, this surplus increased by 21% in the period 2016–2019. The oversupplied specialties include gynecologists, psychiatrists, surgeons, therapists, pediatricians, and radiologists. But at the same time, there is a deficit of hospital resuscitators, ophthalmologists, ultrasound specialists, and psychotherapists (10).

The special program for rural areas has started successfully. In 2012, 7,713 physicians and paramedics settled in rural areas as beneficiaries of the program. However, their influx has slowed down to the level of 5,338 physicians in 2018 (10). Financial benefits work relatively well but their funding is insufficient. The share of recipients of housing and utility allowances in the total number of physicians is only 5.8% (10). An additional limitation is the lack of rural physicians' professional communication with urban medical centers.

The results of the workforce policy, however, are highly dependent on the methodology of the shortage estimates. The official indicator of the occupied positions does not account for multiple job-holding by professionals. This is particularly true for primary care physicians. With a federal norm of 1,700 residents served by each district therapist, the actual average catchment area in 2019 was 2,690 residents, in some regions-−3,000–4,000 (13). According to the survey of physicians, 67% of Russian physicians occupy 1.5 or more positions (14). This phenomenon of multiple job-holding contributes to a substantial gap between the official estimates of the share of occupied positions and the estimates of the actual number of physicians. For example, in Karelia region, the former is 92.8% (nearly all positions are filled), while the latter is only 64.8% (15).

Our estimate of the actual shortage of district therapists, based on the norm of 1,700 adults per physician, is 32%, much higher than the official estimate (13).

While modest gains in reducing the supply shortages of certain types of physicians, the situation of ”too many too few“ has not changed much. The factors that create structural imbalances in the Russian health workforce are still in place.

### Why is the physician shortage reproduced?

#### Service delivery disproportions

They cause the imbalances in the health workforce in the following directions. First, primary care is still the weakest sector of the health system. The task profile of district therapists and pediatricians is limited, they manage only the easiest cases and refer nearly half of patients to outpatient specialists, while their European counterparts manage from 80 to 95% of cases without referrals to specialists (13, 16). The institute of general practitioners with wide clinical and coordinating functions is poorly developed: the share of GPs in the total number of district physicians is only 12.8% and falling (Table 3). This acts as the major driver generating demand for specialists. Meeting this demand is not easy; therefore, the shortage of some specialists is as acute as the deficit of generalists.

Second, a traditional hospital-centered model of service delivery remains in its major features. The number of bed-days per capita is still 70–75% higher than in the EU (6). The work in a hospital is very attractive for the graduates, and their annual influx has generated an oversupply of some hospital doctors. The MoH recognizes this oversupply, but the program does not provide for the redistribution of physicians to polyclinics: the share of outpatient care physicians in the total number of physicians in 2024 will be the same as in 2018, i.e., 55% (Table 1).

Third, the level of physicians' specialization in Russia is very high: there are 92 specialties and subspecialties. Many routine diagnostic tests are performed by specific categories of specialists. Specialization of primary care has reached the point when specialists in polyclinics account for two-thirds of physician positions, while district physicians have lost their primary role. Specialists of polyclinics are usually not involved in inpatient care; therefore, the country needs two categories of specialists—for inpatient and outpatient care. For example, outpatient urologists do not do any surgery. This process of excessive specialization creates demand for an additional number of physicians and increases the number of unfilled positions.

Fourth, the physician shortage is reproduced by a deeply rooted division of labor between physicians and other medical personnel. The nurse-physician ratio in Russia is 2.3 to 1 and is not planned to increase by 2024 (Table 1), while in the USA, Japan, and EU countries, 2.8–4.7 nurses to one physician (1). Nurses' clinical functions are traditionally low in Russia (6). No serious attempts have been made to reduce the demand for physicians by extending the functions of nurses.

#### Health labor planning patterns

Labor imbalances begin with a reliance on weak labor forecasting and planning systems at the regional and federal levels. A long-term vision of the structure of physicians' specialties is needed. In Western countries, in the early 2010s, there were long-term plans for the demand and supply of physicians and nurses in 2030 and even later. These plans were based on the assessment of epidemiological, socio-demographic, and technological factors (17). In Russia, such plans are unavailable. Post-graduate students' enrollment is based on the current assessment of the unfilled positions with a high probability of the graduates' supply not matching demand for specific specialties in the period of 8–10 years.

Regional target enrollment increases the responsibility of regional governments for the employment of graduates but does not reduce the probability of future disproportions across physician specialties. Contracting with medical universities is based on the estimate of current needs, rather than a strategic understanding of future demand and supply for the coming decades. Our analysis of the websites of several regional health authorities shows that not a single region posted estimates of the long-range need for personnel, broken down by individual medical specialties.

The federal MoH has developed a planning methodology, which is based on health care utilization and the number of physicians and nurses per unit of the volume of care (8). But this methodology also suffers from its focus only on current supply and distribution needs. Furthermore, the focus on utilization often provides distorted estimates. For example, a decreasing number of visits to PHC physicians per resident (the recent trend in Russia) results in a decreasing need for the number of primary care physicians. This is contrary to the current objective to strengthen primary care. Other supply factors not addressed include patterns in demographic, trends in disease incidence, and general labor conditions for housing, income, and lifestyle.

#### Inadequate regulation of physicians post-graduate training

The federal MoH develops quotas for the annual admission to post-graduate training in individual specialties, which are then distributed among medical universities based on their applications. The biggest quotas are for the specialties in short supply. However, there is a gap between quotas and the actual applications of medical universities (18). The latter are interested in increasing the number of students who pay for their training. These are the students in the most popular specialties of gynecologists, urologists, and dentists who provide services mostly for out-of-pocket payments. A chronic underfunding of medical universities from public sources aggravates the structure of training: it is shifting to specialties with a high “financial return”. Also, medical universities are slow to adjust their capacity to new needs. Only 3 % of them have units for training GPs (19).

## Actionable recommendations

An extensive increase in the number of physicians does not solve the problem of their shortage. It is necessary for federal and regional policymakers *to strengthen the policy focus on the structural parameters of human resources and the elimination of their imbalances*. The experience of many OECD countries provides good examples of such policies. A range of levers is used, including providing incentives to encourage more doctors to choose a general practice and to foster the take-up of certain specialties that are expected to be in short supply in the future; to expand the roles of nurses and other non-physician providers to reduce pressures on physicians. In post-graduate training, there is a search for a new balance between general practitioners and specialists. For example, in France, 48% of medical graduates go to residency in general practice (20). These trends are very relevant to Russia.

Also, the mechanisms to overcome the hospital-centered model of service delivery are needed. We can suggest (a) strengthening control over opening new positions of hospital doctors, (b) increasing the capacity of outpatient departments in hospitals and staffing them with oversupplied doctors, and (c) retraining some specialists to general practitioners.

*Strengthening health labor planning*. First, to develop middle- and long-term plans for 2030 and 2035, respectively. Second, to account for a complex of factors (future morbidity and mortality, changes in medical technology, possible reconfiguration of physicians' and nurses' roles, and shifts in service delivery structure). Third, to use the indicators of shortage that take into account the multiple job-holding and plan its reduction.

*Strengthening regulation of postgraduate training structure*. To overcome the orientation of medical universities to expand training in oversupplied, well-paying ”commercial“ specialties, it is necessary to increase public funding for medical education. The quotas for post-graduate training should be developed not 1.5 years before the start of admission (as it is now), but 3–4 years before. Universities should have time to change their structure to accommodate the growing number of highly wanted professionals. These quotas should be based on the indicators of strategic forecasts and recruitment plans that may extend up to 10 and 20 years in the future.

*Increasing the share of GPs in the total number of physicians* from the current 13% to the average for the “new” EU countries −29% (19) by 2030. Use financial and nonfinancial incentives for doctors who choose general practice. Cancel the current practice of ”simplified accreditation” of primary care physicians and take a course on modern postgraduate training of GPs. This will strengthen the capacity of PHC and decrease the demand for specialists.

## Conclusion

The health workforce policy in Russia has recently been activated to overcome the shortage of physicians in the situation of serious labor imbalances. Physicians training is expanding, the regions of the country are increasingly contracting medical universities for post-graduate training of specific specialists, and some financial incentives are used. However, the severity of the problem remains high, mostly in primary care. The main reasons for the reproduction of physicians' deficit are the following: a distorted structure of service provision, the lack of medium- and long-term labor planning, the insufficient regulation of post-graduate training structure across specialties, and the underestimate of the general practitioner's role in reducing demand for outpatient specialists. The major lesson learned is that an extensive increase in the number of physicians without changing their composition does not solve the problem. A structural policy is needed to ensure that the workforce structure is in line with the needs of the health system for the coming decade.

## Author contributions

The author confirms being the sole contributor of this work and has approved it for publication.

## Funding

The article was prepared in the framework of a research grant funded by the Ministry of Science and Higher Education of the Russian Federation (Grant ID: 075-15-2022-928).

## Conflict of interest

The author declares that the research was conducted in the absence of any commercial or financial relationships that could be construed as a potential conflict of interest.

## Publisher's note

All claims expressed in this article are solely those of the authors and do not necessarily represent those of their affiliated organizations, or those of the publisher, the editors and the reviewers. Any product that may be evaluated in this article, or claim that may be made by its manufacturer, is not guaranteed or endorsed by the publisher.
